# Research on the estimation and spatial pattern of net tourism carbon emissions in the Yellow River Basin from 2009 to 2019

**DOI:** 10.1007/s11356-024-31902-4

**Published:** 2024-01-17

**Authors:** Ruijuan Peng, Rui Su, Wanqianrong Gao, Xinhong Zhang

**Affiliations:** 1 Tourism College, Northwest Normal University, Lanzhou, 730070 China; 2 Gansu Tourism Development Academy, Lanzhou, 730070 China; 3 School of Design Art, Lanzhou University of Technology, Lanzhou, 730050 China; 4 School of Economics and Management, Anyang University, Anyang, China

**Keywords:** Tourism carbon emissions, Tourism carbon carrying, Capacity, Temporal and spatial evolution, The Yellow River Basin

## Abstract

Based on panel data and remote sensing data of cities in the Yellow River Basin in China from 2009 to 2019, and using the tourism carbon footprint and tourism carbon carrying capacity models, the tourism carbon emissions, tourism carbon carrying capacity, and net tourism carbon of 65 cities in the Yellow River Basin were calculated. The balance and dynamic changes in carbon emissions and carbon fixation of urban tourism in the past ten years were compared. The results show that (1) tourism carbon emissions in the Yellow River Basin are generally on the rise, along with a distribution characteristic of downstream > middle reaches > upstream with obvious characteristics of urban agglomeration centrality within the basin; (2) the carbon carrying capacity of tourism is higher than that of tourism. The growth of carbon emissions is relatively slow, showing a spatial distribution pattern of high in the west and low in the east, which is mainly related to the geographical environment and economic development of the city; (3) the tourism carbon emissions and tourism carbon carrying capacity in the upstream areas can basically maintain a balance, but in the middle and lower reaches of the region, they show a carbon surplus. There is a significant positive spatial correlation in urban net tourism carbon emissions, and the clusters are mainly H-H and L-L.

## Introduction

Global climate change is an important environmental proposition related to the sustainable development of human beings. The Intergovernmental Panel on Climate Change (IPCC 2014) noted in “Climate Change 2014: Synthesis Report” that since the 1950s, the climate system has undergone unprecedented changes. From 1901 to 2010, the average global sea level rose by 0.19%, which had enormous impacts on human and natural systems. Since 2010, China has become the world’s second-largest economy after the United States (Wang and Jiang 2019), and its economic development has attracted worldwide attention. However, China’s previous extensive growth pattern has brought about a series of problems related to resources and the environment (Liang and Yang 2019; Fan et al. 2019). Currently, China is the world’s largest carbon emitter (Liu et al. 2022a), and the large-scale total carbon emissions have placed enormous pressure on the Chinese government to reduce emissions. In the face of many crises and challenges caused by climate change, the Chinese government has been actively fulfilling its corresponding emission reduction obligations and responsibilities. At the 75th session of the United Nations General Assembly, China proposed that carbon dioxide (CO_2_) emissions should peak before 2030 and that China strives to achieve carbon neutrality before 2060 (Yang and Liu 2023; Liu et al. 2022b). This is of great significance in pushing countries around the world to take more decisive actions and promoting global joint efforts to tackle climate change.

Given the rise of global climate change research, tourism, which was once considered a “green industry” and “smoke-free industry,” has become a major source of greenhouse gas emissions (Sun et al. 2020). At the same time, the relationship between tourism and climate has developed from the restrictions on climate with respect to tourism and the need for tourism to adapt to climate change to the current impact of tourism consumption and tourism carbon emissions on climate. The United Nations World Tourism Organization (UNWTO 2009) noted at the Second International Conference on Tourism on Climate Change that tourism contributes approximately 5% to global CO_2_ emissions. However, the research of Liu et al. (2017) shows that China’s tourism carbon emissions account for only approximately 3% of the country’s carbon emissions, which is far from the level of international tourism carbon emissions. Therefore, accurate measurement of the tourism industry carbon emissions is the key to the development of low-carbon tourism in China.

Research on tourism carbon emissions mainly appeared after the 21st century. After Gössling (2002) first proposed to calculate the energy consumption and CO_2_ emissions of the global tourism industry based on transportation, accommodation, and activities, more scholars began to study the tourism industry carbon emissions. In 2006, Becken and Patterson (2006) introduced both “bottom-up” and “top-down” approaches to estimate CO_2_ emissions from tourism in New Zealand. In 2013, Munday et al. (2013) studied the carbon footprint associated with tourism spending in Wales, UK. In current studies, the carbon emission accounting methods of tourism mainly include the “bottom-up” method (Lin 2010), ecological carbon footprint method (Martín-Cejas and Sánchez 2010), and input–output method (Lenzen et al. 2018). Compared with foreign studies, Yang et al. (2008) measured tourism carbon emissions in Shangri-La, Yunnan in 2008. It was not until 2011 that Wu and Shi (2011) used the “bottom-up” method to measure the carbon emissions of China’s tourism industry. China’s research on tourism carbon emissions started late, and the research area mainly involves the national (Meng et al. 2017) and municipal levels (Liu et al. 2011). In general, although there are many studies on tourism carbon emissions in different regions, there is still a lack of comparative research on the tourism carbon footprint and tourism carbon carrying capacity at the watershed scale (Wang et al. 2017).

The Yellow River Basin is an ecological corridor connecting the Qinghai-Tibet Plateau, the Loess Plateau, and the North China Plain. It is an important economic corridor for the construction of the “Belt and Road” initiative and an important link covering and radiating economic and social development in the east, middle, and west (Guo et al. 2020). Studying the relationship between tourism carbon emissions and carbon carrying capacity in the Yellow River Basin plays an important role in proposing differentiated emission reduction paths for local governments. At present, there is insufficient research on the balance and dynamic changes in carbon emissions and carbon fixation in urban tourism in the Yellow River Basin. Therefore, we choose 2009, 2014, and 2019 as the time nodes to avoid the impact of the new coronavirus pandemic on the tourism industry from a data accuracy standpoint, taking 65 prefecture-level and above cities in the basin as the research object; with the help of a carbon emission and carbon carrying capacity calculation model and spatial autocorrelation analysis, we discussed the tourism carbon emissions, carbon carrying capacity, and net tourism carbon in the Yellow River Basin considering the spatial and temporal distribution characteristics of emissions. To enrich the research on tourism carbon emissions in the Yellow River Basin and promote the high-quality and sustainable development of tourism, this study provides a theoretical basis for local governments to formulate low-carbon development policies.

The rest of the paper is structured as follows: “Study area, data sources, and methodology” introduces an overview of the study area, data sources, and research methods. “Results” systematically integrates the calculation results. “Conclusions and discussion” presents the conclusion and discussion and proposes countermeasures and suggestions.

## Study area, data sources, and methodology

### Study area

The Yellow River originates from the northern foot of Bayan Har Mountain in Qinghai Province, with a total length of 5464 km and a total drainage area of approximately 79.5 × 104 km^2^. It is the second-largest river in China (Liu et al. 2022c). The Yellow River Basin is extremely rich in cultural and tourism resources, with 20 world heritage sites, 3497 national A-level tourist attractions, 329 key rural tourism villages, and several national key ecological function zones. In 2019, the Yellow River Basin received 480 million tourist trips and nearly 400 billion yuan in tourism revenue, thus leading tourism to develop into a pillar industry (Zhang et al. 2022). As one of the birthplaces of ancient civilizations, the Yellow River Basin is an important and difficult area for China’s ecological environment security and social and economic development. Conflicts such as fragile natural ecological environments, soil erosion, and water resource shortages are still prominent (Wang et al. 2008). At the same time, there is a large gap in the internal development of the Yellow River Basin, and compared with other coordinated development areas, the overall social and economic development of the Yellow River Basin still lags (Chen et al. 2020).

**Fig. 1:**
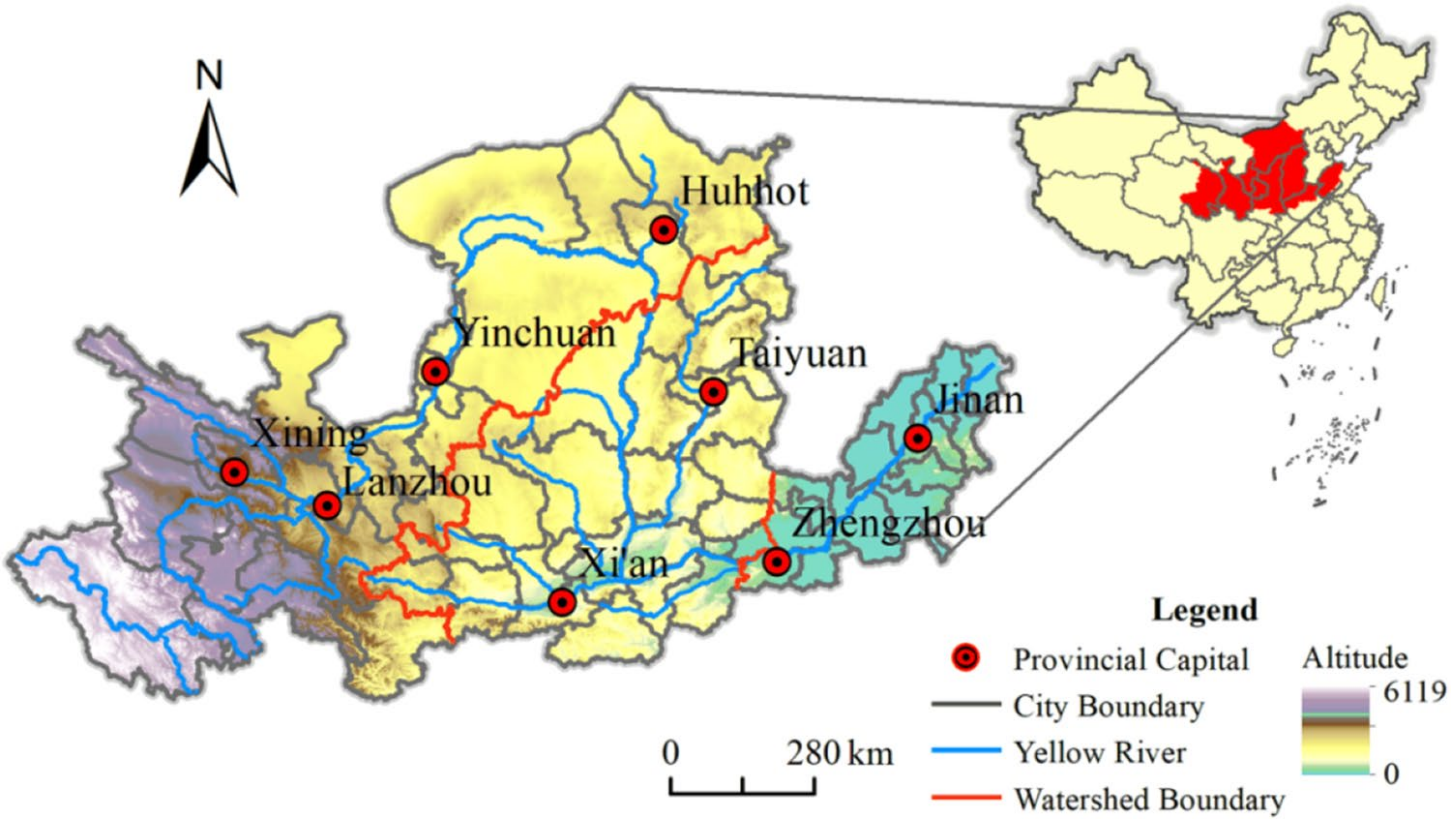
Overview of the study area

Based on the availability of data, 65 prefecture-level cities, including Qinghai, Gansu, Ningxia, Inner Mongolia, Shanxi, Shaanxi, Henan, and Shandong, were selected as the research area of this paper, as shown in Fig. 1. In addition, referring to related research (Jiang et al. 2022), the Yellow River Basin is divided into upper reaches (Qinghai, Gansu, and Ningxia), middle reaches (Shaanxi, Shanxi, and Inner Mongolia) and lower reaches (Henan and Shandong); see Table 1.

**Table 1:** Cities included in each section of the Yellow River Basin

Watershed segment	Province	City
Upper	Qinghai	Xining, Haidong, Huangnan Tibetan Autonomous Prefecture, Hainan Tibetan Autonomous Prefecture, Haibei Tibetan Autonomous Prefecture, Golog Tibetan Autonomous Prefecture
	Gansu	Lanzhou, Baiyin, Dingxi, Tianshui, Pingliang, Qingyang, Longnan, Linxia Hui Autonomous Prefecture, Gannan Tibetan Autonomous Prefecture
	Ningxia	Yinchuan, Shizuishan, Wuzhong, Zhongwei, Guyuan
Middle	Shaanxi	Xi’an, Tongchuan, Baoji, Xianyang, Yan’an, Yulin, Weinan, Shangluo
	Shanxi	Taiyuan, Datong, Yangquan, Changzhi, Jincheng, Shuozhou, Jinzhong, Yuncheng, Xinzhou, Linfen, Lvliang
	Inner Mongolia	Huhehaote, Baotou, Wuhai, Ordos, Bayan Nur, Ulaanchabu
Lower	Henan	Zhengzhou, Kaifeng, Luoyang, Anyang, Hebi, Xinxiang, Puyang, Sanmenxia, Shangqiu, Jiaozuo, Jiyuan
	Shandong	Jinan, Zibo, Dongying, Jining, Tai’an, Dezhou, Liaocheng, Binzhou, Heze

### Data sources

The research object selected in the article is 65 cities in the Yellow River Basin, and the sample years are 2009, 2014, and 2019 for a total of three time sections. The data sources of this article mainly include (1) vector administrative district boundary data from the National Center for Basic Geographic Information (http://www.ngcc.cn), (2) DEM data from the Geospatial Data Cloud (https://www.gscloud.cn), (3) land use classification data with a 1 km resolution selected from the Chinese Academy of Sciences Resource and Environmental Science Data Center (http://www.resdc.cn), and (4) social and economic data from the “China City Statistical Yearbook” (http://www.stats.gov.cn) and the statistical bulletins of the national economic and social development of various provinces and cities (from the official websites of the statistical bureaus of each province and city, such as the National Economic and Social Development of Gansu Province and Social Development Statistical Bulletin: https://tjj.gansu.gov.cn).

### Methodology

#### Tourism carbon emission measurement

Tourism carbon emissions (TCE) are the greenhouse gasses produced by tourists in the process of consuming tourism products. Referring to current research (Cao et al. 2014), the calculation formula of tourism carbon emissions in this paper is as follows:

1 $$\textrm{TCE}=A\times G$$

where TCE denotes urban tourism carbon emissions in tens of thousands of tons, *A* denotes urban tourism revenue in thousands of yuan, and *G* represents the CO_2_ emission intensity of urban tourism. Since there are no relevant data on the carbon emission intensity coefficient of China’s tourism industry, we refer to the research results of Dong et al. (2018) and Tu and Liu (2021) and find that the world average tourism carbon emission intensity coefficient is 623, using 13 kg/thousand dollars (global carbon emission intensity is 92.9–835.8 kg/thousand dollars, and the intensity of tourism carbon emissions is high) as a reference value (the unit is converted into kg/yuan according to the exchange rate of the RMB and US dollars in the year).

#### Tourism carbon carrying capacity measurement

**Fig. 2:**
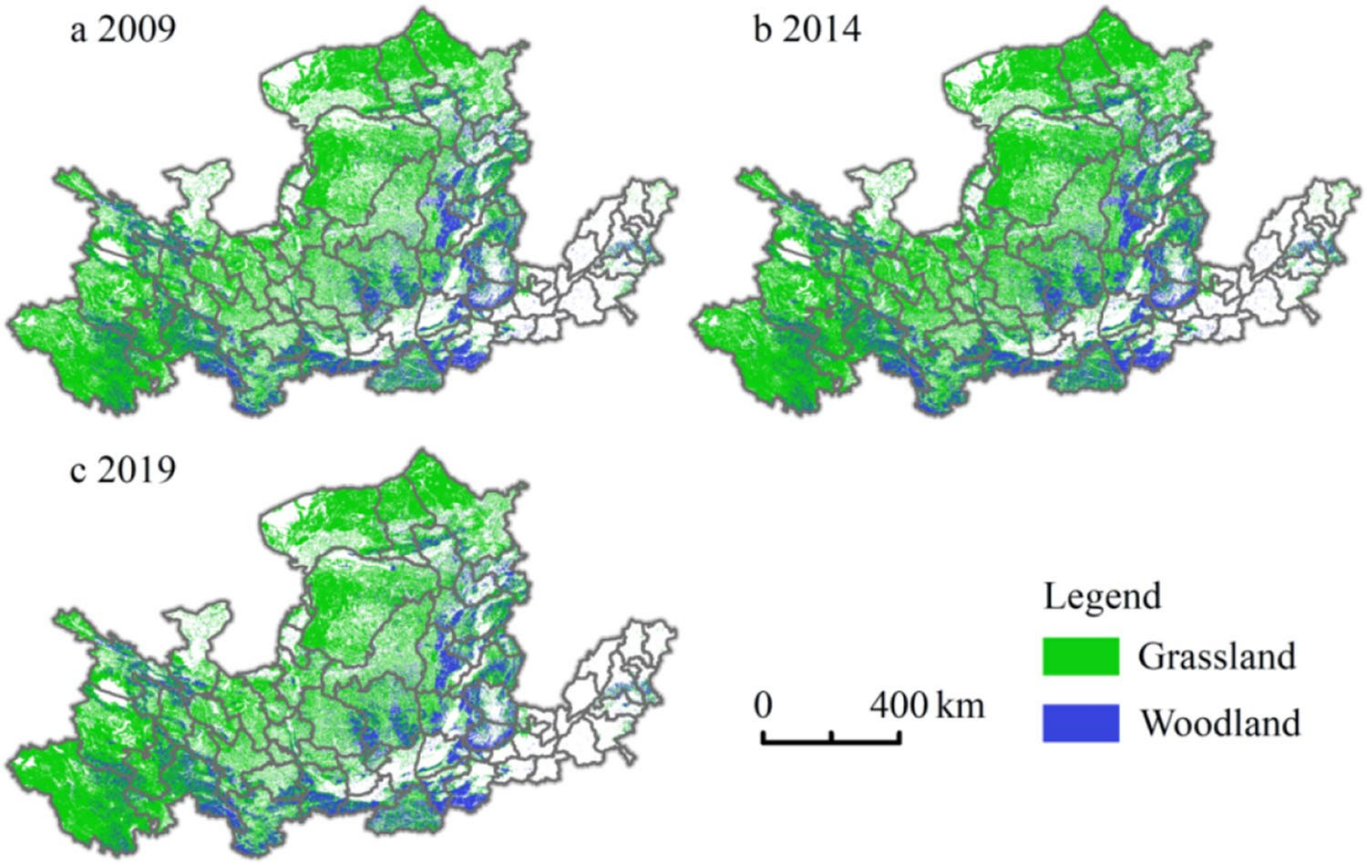
Spatial distribution of grassland and woodland in the Yellow River Basin

The carbon carrying capacity (TCC) refers to the maximum amount of carbon dioxide absorbed by vegetation in a region through photosynthesis and refers to the carbon sink capacity of vegetation. In this paper, tourism carbon carrying capacity is defined as the amount of carbon dioxide from tourism fixed by photosynthesis in the ecosystem in the region. Referring to the study of Ren et al. (2019), the calculation formula of tourism carbon carrying capacity in this paper is as follows:

2 $$\textrm{TCC}=\textrm{CC}\times r$$

3 $$\textrm{CC}=\sum {A}_i\times {\textrm{NEP}}_i\times 44/12$$

4 $$r={\textrm{GDP}}_t/\textrm{GDP}$$

where TCC denotes the carbon carrying capacity of urban tourism, in tons; CC denotes the total carbon carrying capacity of the city, in tons; *r* denotes the coefficient of urban carbon carrying capacity; GDP_*t*_ and GDP denote the total tourism income and gross local product of the city, in billion yuan, respectively; *i* denotes the type of vegetation; considering the characteristics of vegetation in the study area, the vegetation selected in this paper includes forest and grassland; *A*_*i*_ denotes the type *i* area of vegetation in hectares, obtained through land use data, as shown in Fig. 2; NEP_*i*_ denotes the net ecosystem productivity of vegetation of type *i* in tons/ha/year, referring to the study of Zhao et al. (2015); the NEP values of grassland and woodland were taken as 0.94 and 3.81, respectively; and 44/12 denotes the conversion rate of carbon dioxide to carbon.

#### Net tourism carbon emission measurement

In this paper, the difference between tourism carbon emissions and tourism carbon carrying capacity is defined as net tourism carbon emissions (NTCE), which can be used to determine whether a region is in a state that promotes or limits climate warming. The calculation formula is as follows:

5 $$\textrm{NTCE}=\textrm{TCE}-\textrm{TCC}$$

where NTCE is the net tourism carbon emission in million tons. When NTCE < 0, it means that the city tourism carbon emission is carbon deficit, and the current tourism activities of the city are environmentally friendly and in line with the concept of sustainable development. When NTCE > 0, it means that the city’s tourism carbon emissions are in carbon surplus, and the tourism activities carried out by the city have increased the burden of the ecosystem in the region.

#### Spatial autocorrelation analyses

Spatial autocorrelation analysis can be divided into global spatial autocorrelation analysis and local spatial autocorrelation analysis, which explains the overall distribution of specific phenomena and whether these phenomena present clustering characteristics in a given space commonly using Moran’s index (Moran’s *I*) measure (Wang et al. 2019; Zhang et al. 2020). Therefore, this paper uses Moran’s *I* to explore and analyze the spatial data of net tourism carbon emissions.

The calculation formula of the global Moran’s *I* index is as follows:

6 $$I=\frac{n\times {\sum}_i^n{\sum}_j^n{W}_{ij}\left({x}_i-\overline{x}\right)\left({x}_j-\overline{x}\right)}{\sum_i^n{\sum}_j^n{W}_{ij}\times {\sum}_i^n{\left({x}_i-\overline{x}\right)}^2}$$

where *n* is the number of spatial units indexed by *i* and *j*, *x* is the variable of interest, *x*_*i*_ and *x*_*j*_ are the values of the observed variable at sites *i* and *j*, $\overline{x}$ is the mean of *x*, the weights *W*_ij_ are written in a (*n* × *n*) weight matrix, and the weight matrix depicts the relation between an element and its surrounding elements. Weight can be based on contiguity relations or distance. The value of Moran’s *I* is between −1 and 1. When Moran’s *I* is greater than 0, it indicates positive spatial correlation. The larger the value, the more obvious the spatial correlation is. The smaller the space, the greater the difference; when Moran’s *I* is equal to zero, the space is random.

The calculation formula of the local Moran’s *I* index is as follows:

7 $${I}_i={Z}_i^{\prime }{\sum}_i^n{W}_{ij}{Z}_i^{\prime }$$

where ${Z}_i^{\prime }$ and ${Z}_j^{\prime }$ are the original variables of xi and xj in standardized forms, respectively, and *W*_ij_ is the spatial weight matrix. Through the measured local Moran index, we can obtain the LISA scatter diagram we need. The scatter diagram can be divided into four quadrants. The first is the agglomeration (HH) with high self-emissions and high emissions in neighboring cities. The first quadrant indicates that the space is positively correlated; then, there is an agglomeration (LH) with low self-emissions but high emissions in neighboring cities, which is in the second quadrant, indicating that the space is negatively correlated; then, it is low within itself and in adjacent cities. Additionally, low agglomeration (LL), which is in the third quadrant, indicates that the space is positively correlated; finally, the agglomeration (HL) with high self-emissions but low emissions of neighboring cities is in the fourth quadrant, and it indicates that the space is negatively correlated.

## Results

### Temporal and spatial variation characteristics of tourism carbon emissions in the Yellow River Basin

**Fig. 3:**
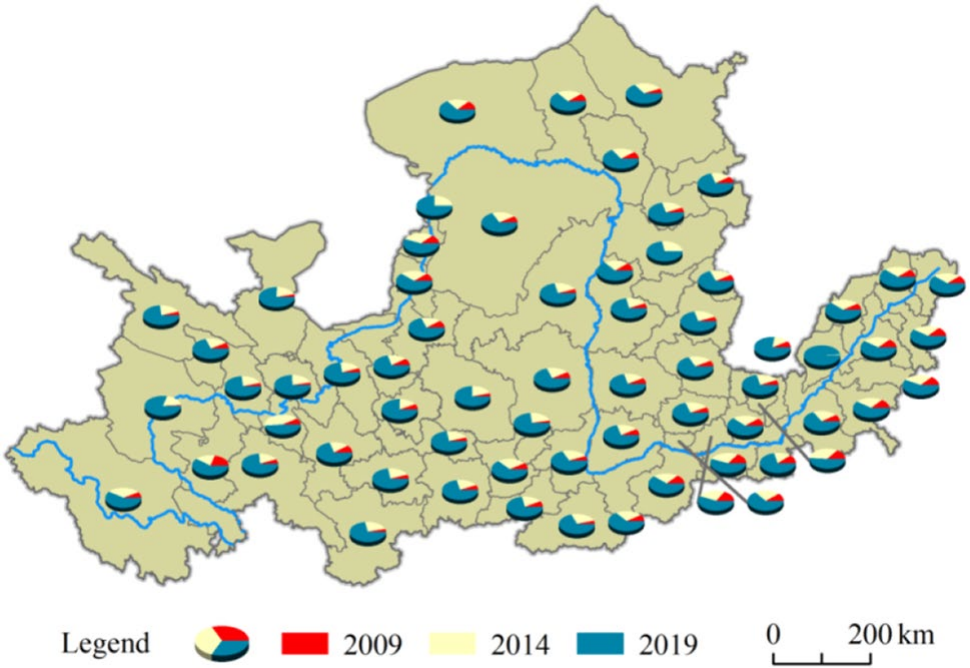
The proportion of tourism carbon emissions in each year of tourism in 65 cities in the Yellow River Basin

From 2009 to 2019, the total tourism carbon emissions of cities in the Yellow River Basin showed a continuous upward trend, increasing from 39.23 Tg (1 Tg = 1 × 10^12^ g) in 2009 to 404.84 Tg in 2019, with an average annual growth rate of 29.61%. The growth rate of tourism carbon emissions from 2009 to 2014 was 205.25%, and the growth rate from 2014 to 2019 was 240.30%, showing a continuous but increasing growth trend, and thus it is consistent with the trend of total tourism carbon emissions. The proportion of carbon emissions in each city in the basin is shown in Fig. 3. In 2009, more than 75% of the cities’ tourism carbon emissions accounted for less than 10% of the total, and most of these cities were located in the middle and upper reaches. The overall tourism carbon emissions were at a low level. In 2014, tourism carbon emissions accounted for 20–30% of the total in more than 78% of cities. In 2019, more than 87% of cities accounted for more than 60% of tourism carbon emissions, which shows that the number of cities with high tourism carbon emissions is also increasing.

**Fig. 4:**
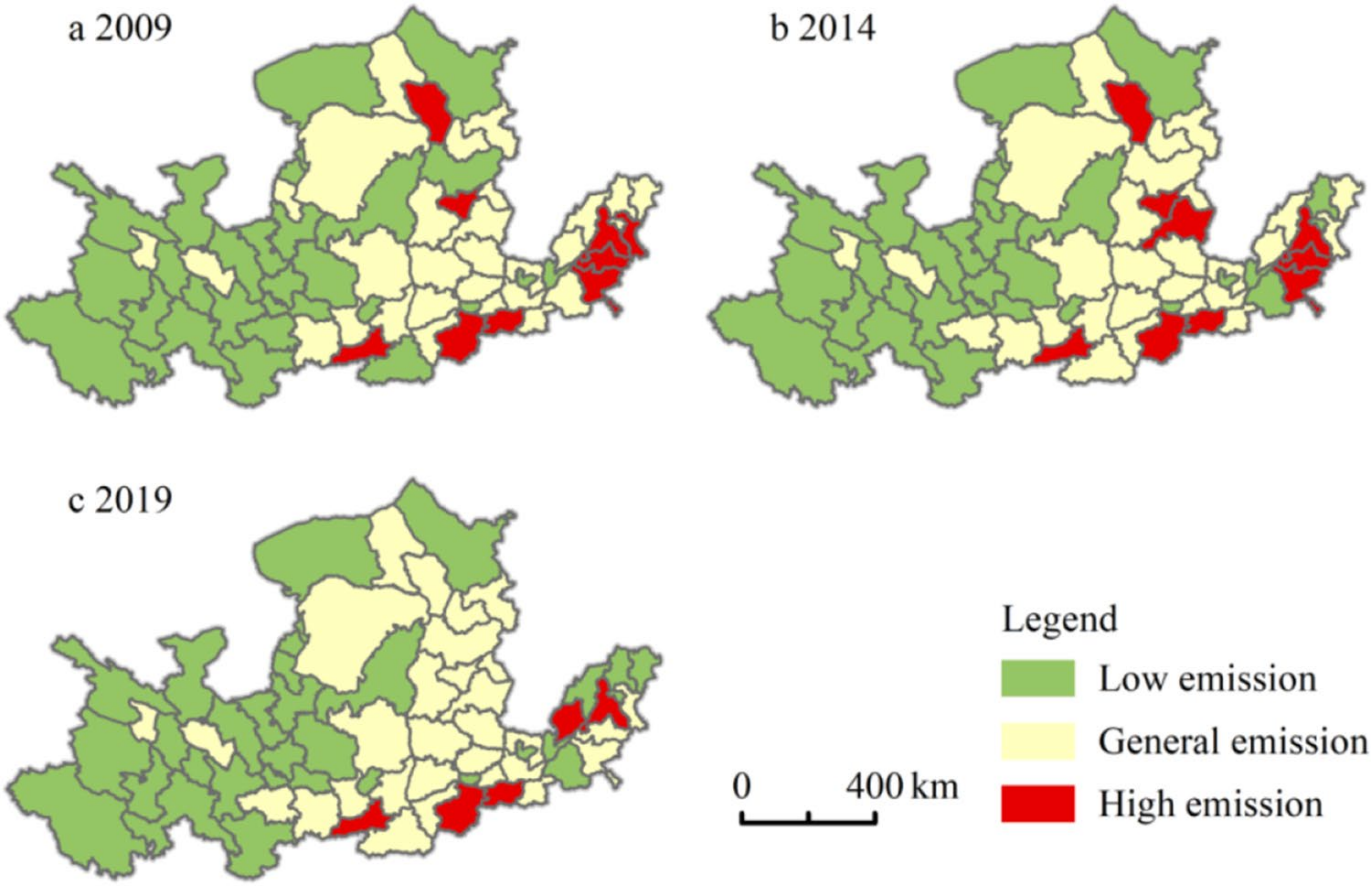
Spatial variation in total tourism carbon emissions in 65 cities in the Yellow River Basin

For further analysis, this paper uses *K*-cluster analysis (Sreedhar et al. 2017) as the basis for grading urban tourism carbon emission intensity. The clustering results are shown in Fig. 4.

The areas with high levels of carbon emissions from tourism in the Yellow River Basin are mainly distributed in the middle and lower reaches of the Yellow River, in the Central Plains urban agglomeration (Zhengzhou and Luoyang), in the Shandong Peninsula urban agglomeration (Jining, Zibo, and Jinan), in the Guanzhong Plain urban agglomeration (Xi’an), and in the Jinzhong urban agglomeration (Taiyuan). The top three high-level carbon emission areas are Xi’an (14.38 Tg) in the Guanzhong Plain urban agglomeration, Zhengzhou (9.46 Tg) in the Central Plains urban agglomeration, and Jinan (7.21 Tg) in the Shandong Peninsula urban agglomeration. The carbon emission intensity of tourism is at a high level, and it has a prominent feature of urban agglomeration centrality.

The number of cities with high levels of tourism carbon emissions showed a decreasing trend, and the spatial distribution of cities with medium and high levels of tourism carbon emissions did not change significantly. The entire tourism carbon emissions in the basin showed the distribution characteristics of downstream > middle > upstream, and the spatial distribution characteristics of tourism carbon emissions were high in the east and low in the west, high in the middle and lower reaches, and low in the upstream.

### Temporal and spatial variation characteristics of tourism carbon carrying capacity in the Yellow River Basin

**Fig. 5:**
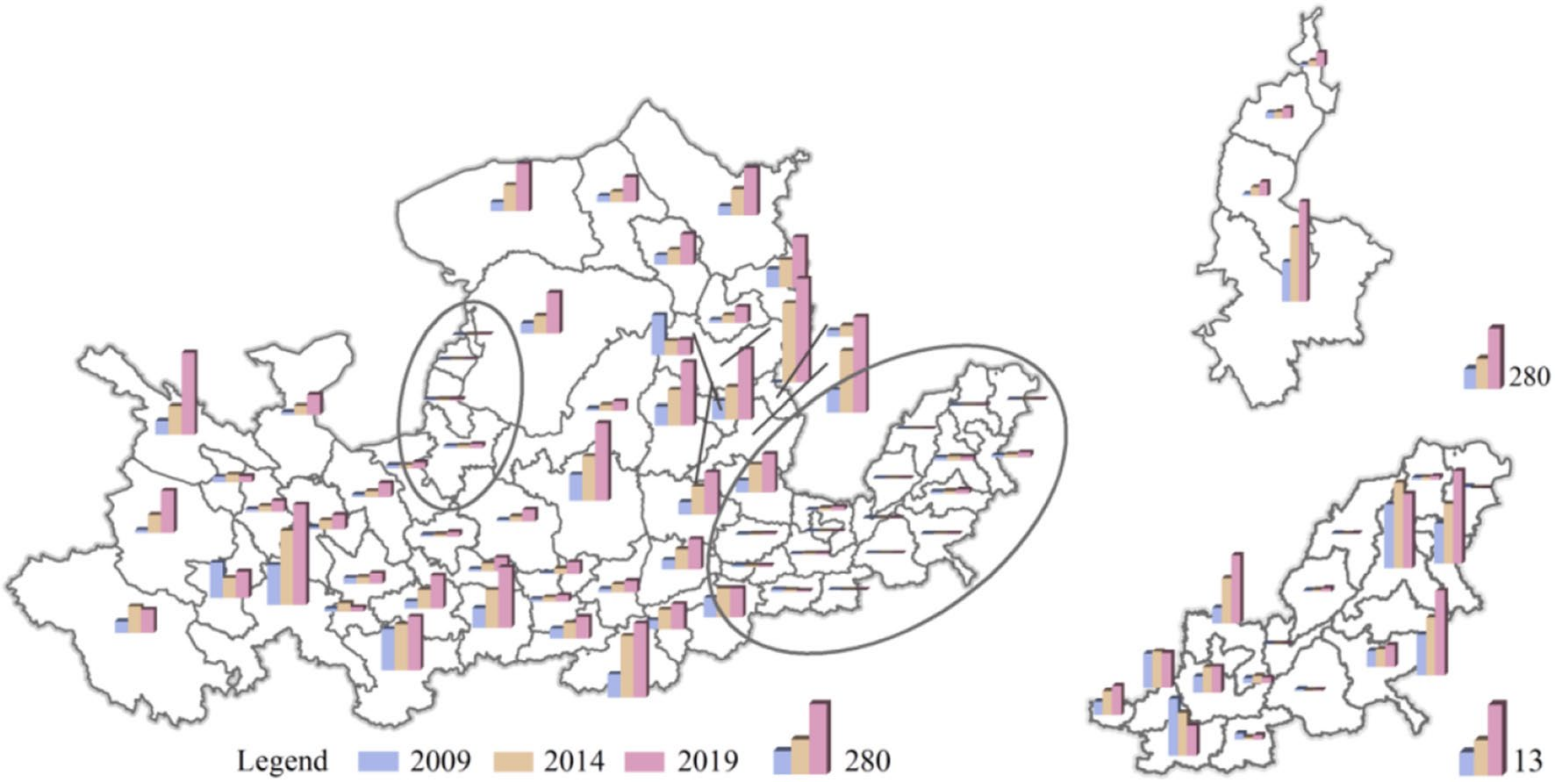
Schematic diagram of changes in the tourism carbon carrying capacity of 65 cities in the Yellow River Basin

The change in tourism carbon carrying capacity in the Yellow River Basin is shown in Fig. 5. The tourism carbon carrying capacity was 29.21 Tg in 2009 and increased to 86.22 Tg in 2019, an increase of 195.17%. In 2019, more than half of the mid-upstream cities had a tourism carbon carrying capacity of more than 50% of the 3-year statistical total, indicating that the mid-upstream cities achieved good results in the practice of carbon emission reduction while vigorously developing tourism. However, the tourism carbon carrying capacity of the Shandong Peninsula urban agglomeration in the lower reaches of the Yellow River has only slightly changed, which is closely related to the economic development and land use of the downstream cities.

**Fig. 6:**
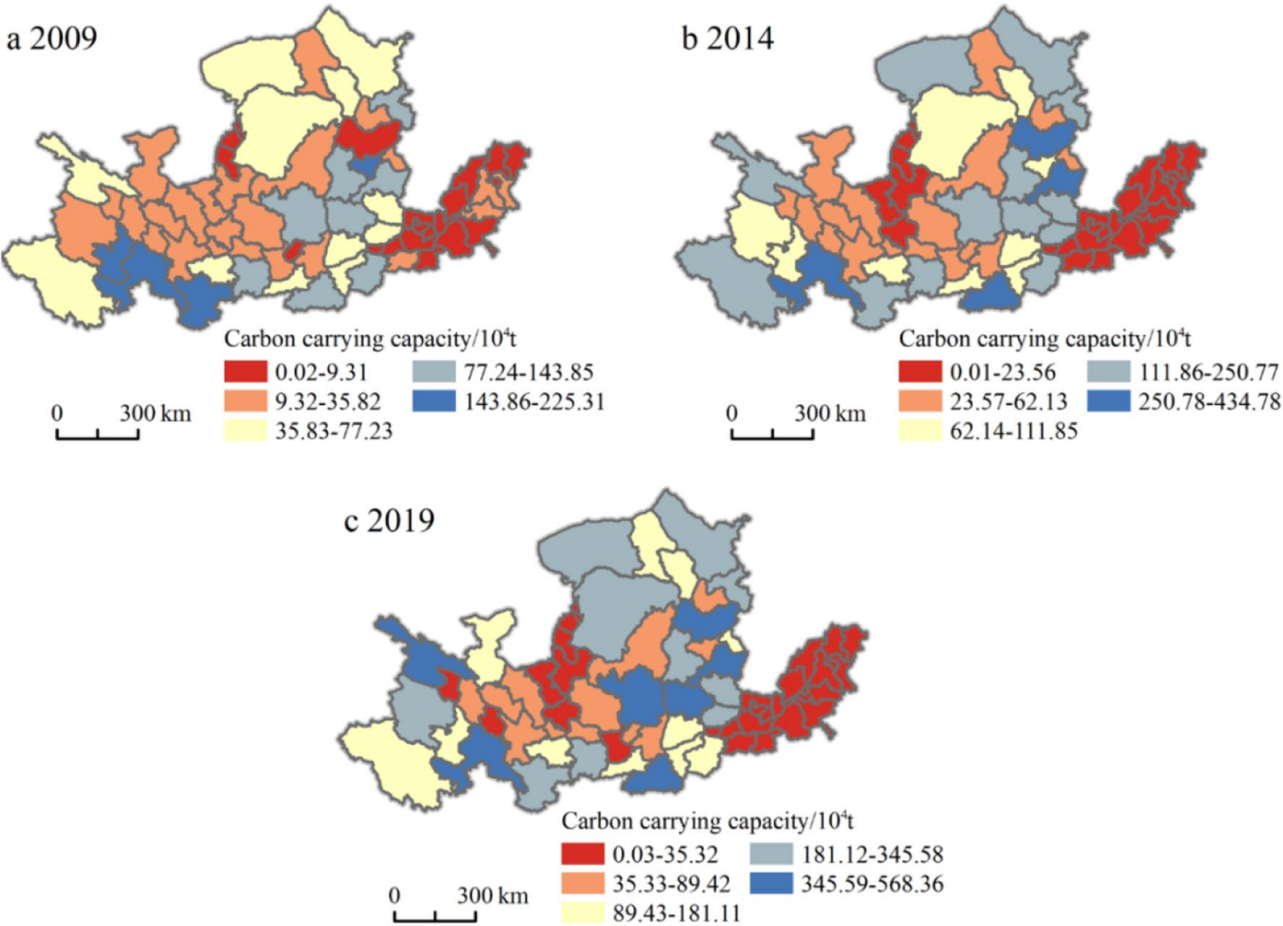
Spatial variation in the total tourism carbon carrying capacity in 65 cities in the Yellow River Basin

The spatial variation in tourism carbon carrying capacity in the Yellow River Basin is shown in Fig. 6. There are large differences in the intensity of tourism carbon carrying capacity in various cities. The areas with high tourism carbon carrying capacity are mainly concentrated in the middle and upper reaches. The top three tourism carbon carrying capacities are Gannan Tibetan Autonomous Prefecture (3.92 Tg), Xinzhou (3.34 Tg), and Jinzhong (3.26 Tg). Compared with the tourism carbon carrying capacity in 2009, with an overall increase of 195.17% in 2019, the upstream and downstream growth rates were 185.49% and 200.25%, respectively, and the midstream cities also showed an obvious upward trend, with an increase of 33.03 Tg and an increase of 222.26%. This is confirmed by Jia’s (2018) research on forest coverage in the Yellow River Basin. Generally, the reasons for the large gap in tourism carbon carrying capacity between cities in the Yellow River Basin can be divided into two categories. One is that the vegetation coverage of the city itself is relatively small, and due to rapid economic development, human activities have a greater impact on the environment. For example, Puyang City, Heze City, Liaocheng City, Dezhou City, etc. show this phenomenon. The other category is due to poor weather conditions and less precipitation, which is not conducive to the growth of grassland and woodland, resulting in a small tourism carbon carrying capacity, such as in Yinchuan City, Wuzhong City, and Guyuan City.

### Temporal and spatial variation characteristics of net tourism carbon emissions in the Yellow River Basin

The total net tourism carbon emissions of the Yellow River Basin in 2009, 2014, and 2019 were 10.01 Tg, 63.74 Tg, and 318.62 Tg, respectively, which means that the overall carbon state is in a state of carbon surplus, and it is showing a trend of rapid growth. This result shows that the carbon emissions due to tourism activities in the Yellow River Basin between 2009 and 2019 were much higher than the carbon sequestration of vegetation, and the ecological pressure of tourism carbon showed a continuous upward trend. From the perspective of watersheds (see Table 2), the high value of tourism net carbon emissions mainly occurs in the middle and lower reaches, and the growth rate is relatively large, while the upper reaches of tourism net carbon emissions changed from carbon deficit to carbon surplus between 2014 and 2019. In recent years, due to the rapid development of tourism in the Yellow River Basin and the rapid increase in the number of tourists, the level of net tourism carbon emissions in the Yellow River Basin has become relatively high and is in a stage of rapid rise. The carbon emission reduction of the tourism industry in the Yellow River Basin is facing severe challenges.

**Table 2:** The net tourism carbon emissions (Tg) of each section of the Yellow River Basin

	2009	2014	2019
Upper	−8.45	−8.16	0.11
Middle	0.76	25.35	111.76
Lower	17.71	46.56	206.74
Total	10.01	63.74	318.61

**Fig. 7:**
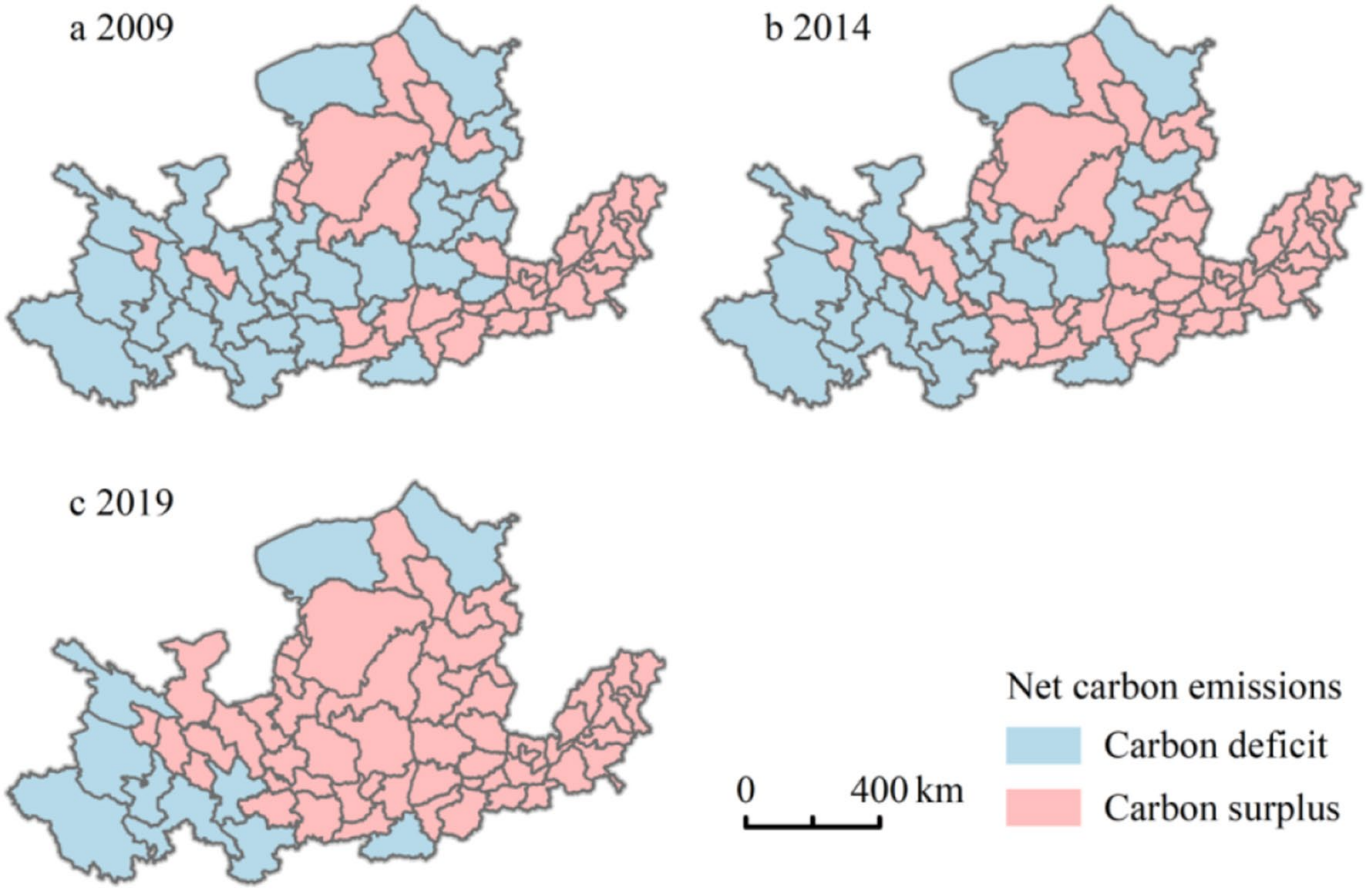
Spatial change in net tourism carbon emissions in 65 cities in the Yellow River Basin

Figure 7 shows the spatial variation in the net tourism carbon emissions of tourism in the Yellow River Basin. In 2014, 30 cities in the middle and upper reaches showed a carbon deficit, and the net tourism carbon emission situation was still good. Among them, Longnan (−2.20 Tg), Gannan Tibetan Autonomous Prefecture (−2.15 Tg), and Huangnan Tibetan Autonomous Prefecture (−1.87 Tg) have the highest carbon sinks. In 2014, 67.69% of the cities showed a carbon surplus, and the scale of the carbon surplus urban agglomeration expanded, adding the Jinzhong urban agglomeration and the Guanzhong Plain urban agglomeration. In 2019, only 10 cities showed carbon deficits. From the perspective of regional distribution, the downstream Shandong Peninsula urban agglomeration was always in a state of carbon surplus from 2009 to 2019. For example, Liaocheng is a typical city with carbon emissions, and the problem of climate and environmental degradation cannot be ignored.

The Moran’s *I* index of the net tourism carbon emissions of the Yellow River Basin was 0.28, 0.17, and 0.29 in 2009, 2014, and 2019, respectively; all figures were greater than zero and passed the 1% significance test, which indicated that the net tourism carbon emissions of the Yellow River Basin had significant spatial autocorrelation, and there was a phenomenon of concentrated distribution of cities with similar carbon emission levels. At the same time, because Moran’s *I* index in 2019 is the largest, the spatial agglomeration of net tourism carbon emissions in 2019 is stronger than that in 2009 and 2014. It showed a trend of first decreasing and then increasing.

**Fig. 8:**
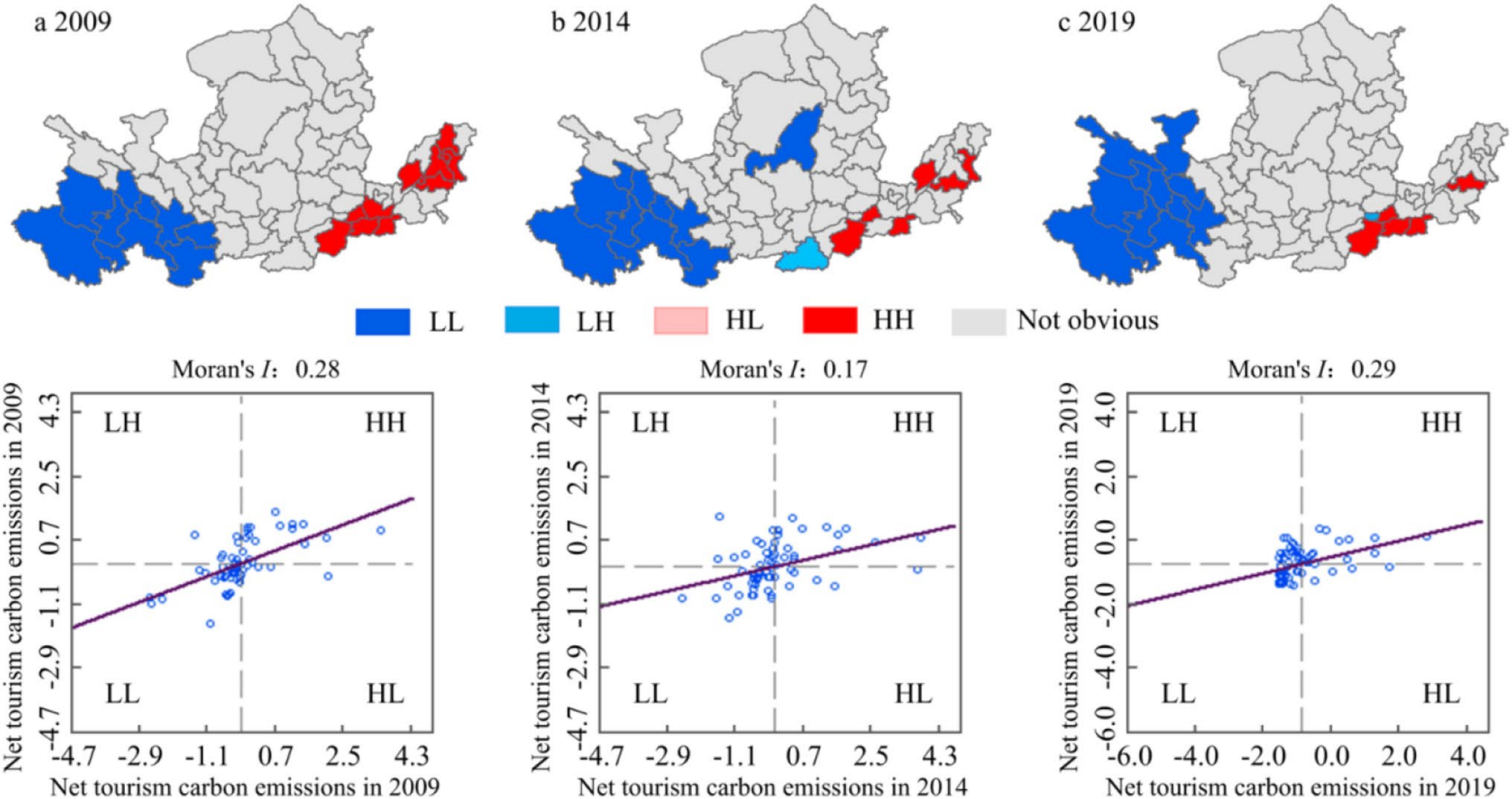
LISA clustering and Moran’s *I* scatter plot of net tourism carbon emissions in the Yellow River Basin

The local agglomeration characteristics of net tourism carbon emissions in the Yellow River Basin were analyzed by a LISA cluster diagram. It can be seen from Fig. 8 that there was no HL-type aggregation distribution type in the three years, and the LH-type aggregation distribution only appeared in 2014 and 2019. There is only one city of this aggregation type. In 2009, the proportions of LL-type and HH-type cities were 16.07% and 17.86%, respectively. By 2019, the proportions of LL-type and HH-type cities were 19.64% and 8.92%, respectively. Compared with 2009, the level of local spatial agglomeration in 2019 increased. The number of LL-type cities increased by 2, while the number of HH-type cities decreased by 5. The spatial agglomeration is changing to LL-type. From 2009 to 2019, the distribution of HH-type and LL-type cities was relatively concentrated. LL-type cities are mainly distributed in the upstream area, while HH-type cities are mainly distributed in the downstream area, and there is no obvious spatial aggregation effect in the middle reaches.

### Per capita net tourism carbon emissions and emission intensity

**Fig. 9:**
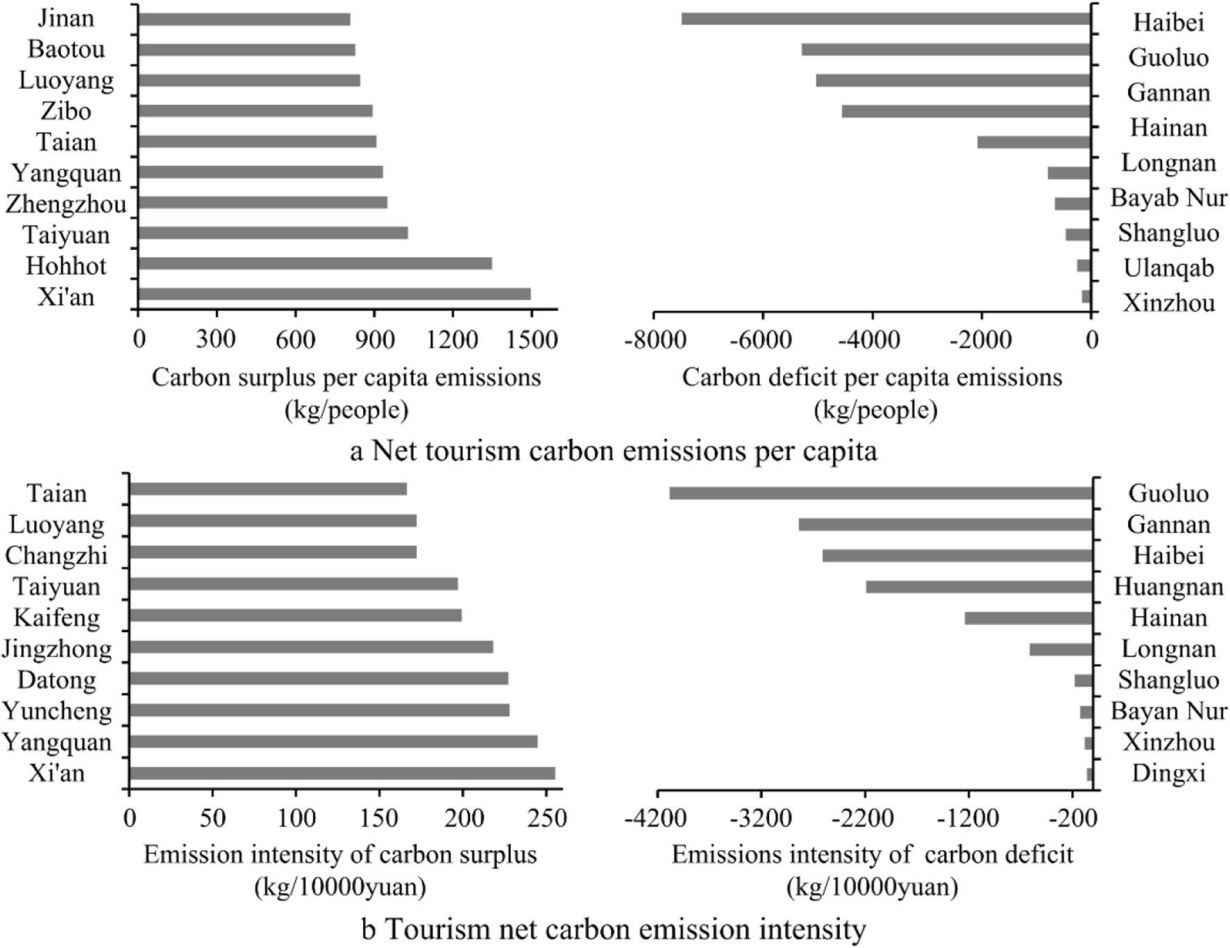
Per capita net tourism carbon emissions and changes in emission intensity in the Yellow River Basin

The ranking of the top ten cities in the Yellow River Basin in terms of per capita net tourism carbon emissions and emission intensity is shown in Fig. 9. Per capita net tourism carbon emissions show a change characteristic of being higher in the middle and lower reaches than in the upstream. Looking at cities with carbon surplus, the average per capita net tourism carbon emissions in the top ten cities is 1004.33 kg/people. Among them, Xi’an, located in the middle reaches of the Yellow River, has the highest per capita emissions, followed by Hohhot, both of which have per capita emissions of more than 1200 kg/ people. The remaining top ten cities have maintained around 900 kg/ people. Looking at cities with carbon deficits, the cities with the smallest net carbon emissions per capita are all from the upper reaches of the river basin. Although the sparse population in these areas is not conducive to reducing local per capita carbon emissions, the good local ecological environment has become a good source of carbon absorption. Main places are keeping per capita net carbon emissions below −4000 kg/ people.

Emission intensity refers to the net tourism carbon emissions per unit of GDP. Its changing characteristics are similar to per capita emissions. High-value areas mainly appear in the middle and lower reaches of the basin, while the upstream still shows a relatively obvious carbon deficit. Among cities showing a carbon surplus, the average emission intensity is 110.62 kg/10,000 yuan, of which the top ten cities are all greater than 160 kg/10,000 yuan. Xi’an ranks first with an emission intensity of 255.21 kg/10,000 yuan. Among the cities showing a carbon deficit, upstream cities are still the main ones. This is mainly due to the lagging economic development of Guoluo, Gannan, and other places, which results in a larger carbon absorption per unit of GDP. The emission intensity of Guoluo reaches −4084.8 kg/10,000 yuan, which is 1.4 times that of Gannan, which ranks second.

## Conclusions and discussion

### Conclusions

This paper first calculates the tourism carbon emissions and tourism carbon carrying capacity of 65 prefecture-level and above cities in the Yellow River Basin from 2009 to 2019, uses the difference between the two to obtain the net tourism carbon emissions, analyzes their temporal and spatial evolution characteristics, and finally analyzes the spatial clustering characteristics of net tourism carbon emissions with the help of Moran’s *I*. The following research conclusions are drawn.

1. From 2009 to 2019, tourism carbon emissions in the Yellow River Basin showed an increasing trend, with an average annual growth rate of 29.61%. Tourism carbon emissions decreased in the order of downstream > midstream > upstream, showing the spatial distribution characteristics of high in the east and low in the west, high in the middle and lower reaches, and low in the upstream. In addition, the number of cities with high tourism carbon emissions is increasing, and they show significant characteristics of urban agglomeration centrality
2. The tourism carbon carrying capacity of the Yellow River Basin also showed an increasing trend from 2009 to 2019, but its average annual growth rate was 12.77%, which was lower than the average annual growth rate of carbon emissions. There are large differences in the intensity of tourism carbon carrying capacity among cities, and the areas with high tourism carbon carrying capacity are mainly concentrated in the middle and upper reaches. The reasons for this difference can be summarized in two categories. The other category is that the city itself is in a harsh natural environment
3. Only the upper reaches of the Yellow River Basin had carbon deficits in 2009 and 2014, and most of these cities were regions with lagging tourism development, while other regions and years had carbon surpluses exhibiting greater pressure to reduce emissions. In addition, through Moran’s *I* analysis, it is found that the net tourism carbon emissions in the basin have significant spatial autocorrelation, and there is a phenomenon of concentrated distribution of cities with similar carbon emission levels. The upper reaches of the watershed are dominated by LL-type aggregation distribution, the lower reaches are dominated by HH-type aggregation, and the middle reaches have no significant spatial aggregation effect

### Discussion

As a strategic pillar industry for the development of the national economy, tourism has brought enormous economic benefits, but its negative effects on the ecological environment have become increasingly obvious. The Yellow River Basin straddles the east, middle, and west regions and is an important economic belt and ecological barrier in China. Exploring the temporal and spatial variation characteristics of tourism carbon emissions in the Yellow River Basin can promote the realization of the overall high-quality development of the Yellow River Basin from the perspective of tourism. Based on the calculation of tourism carbon emissions and tourism carbon carrying capacity of the Yellow River, this paper further analyzes the temporal and spatial variation characteristics of net tourism carbon emissions, which is of great significance for the precise formulation of emission reduction measures in different regions.

The emission sources involved in tourism carbon emissions are relatively complex (Liu et al. 2022a), mainly because most of the products and services consumed by tourists are not limited to tourism (Neger et al. 2021), and carbon emission estimates cannot take into account the entire consumption process, which brings great uncertainty to the estimated results. This paper uses the world average tourism carbon emission intensity to calculate carbon emissions, and the calculation results are basically consistent with various current studies (see Table 3). Although this method has been adopted by most studies, there are certain limitations. The research of Becken and Simmons (2002) showed that 65–73% of the total energy consumption of the tourism industry comes from tourism traffic, which verified that the main source of carbon emissions in the tourism industry is tourism traffic. Therefore, a sound tourism statistical system is crucial to the study of tourism carbon emissions.

**Table 3:** Tourism carbon emissions (Tg) of some existing studies in the Yellow River Basin

Source	Yuan et al. (2022)	Luo et al. (2020)	Huang and Tang (2021)	This study		
	2017	2010	2009	2009	2014	2019
Carbon emission	382.2	47.53	26.00	39.23	118.96	404.84

In the future, we can further analyze the factors affecting tourism carbon emissions and predict their development trends to determine the energy-saving and emission reduction potential of tourism carbon emissions and provide government departments with more targeted, more scientific, and effective decision-making by quantitatively describing them. This is of great significance for the further development of low-carbon tourism and the promotion of ecological civilization in the Yellow River Basin.

### Policy implications

Based on the above conclusions, this paper puts forward the following policy recommendations.

First, optimize the energy structure and improve utilization efficiency. The research results show that tourism carbon emissions in the Yellow River Basin are far greater than its carbon carrying capacity, and the carbon imbalance in tourism is significant. Therefore, local governments should accelerate the development of green energy such as electricity and solar energy, increase investment in scientific research, and improve the tourism industry and green innovation environment. Actively develop and introduce low-carbon energy-saving technologies, improve energy utilization efficiency, and promote the green transformation of energy in tourism-related industries, for example, by implementing preferential policies for the purchase of new energy vehicles for tourism companies, rationally planning energy supply stations and other measures to promote electric vehicles and solar vehicles, and using as many green energy facilities and equipment as possible in the transportation industry.

Second, the concept of green consumption should be cultivated, and enterprises should be urged to manage themselves. Tourists are the main body of tourism activities, and formulating and issuing green tourism consumption guidelines, strengthening public welfare publicity, and cultivating tourists’ green consumption concepts can effectively promote energy conservation and emission reduction in the tourism industry. In addition, the concept of green travel can also be integrated into the compulsory education to cultivate children’s green consumption concept from an early age and drive the formation of the whole family’s green consumption concept through children. Tourism enterprises, as providers of tourism products and services, should systematically improve the level of green innovation in all stages of product development and design, production, and manufacturing and further guide tourists to green consumption. Tourism management departments should formulate and improve green tourism conventions suitable for local development as soon as possible and urge tourism enterprises to realize green transformation.

Third, a horizontal carbon compensation mechanism should be built for the tourism industry of the urban agglomeration in the Yellow River Basin. All provinces should breakdown administrative barriers; strengthen the exchange of experience in carbon emission reduction; set up a special research group on carbon compensation for tourism in the Yellow River Basin; formulate horizontal carbon compensation specifications for tourism that are suitable for the characteristics of the urban agglomeration in the Yellow River Basin; build a horizontal carbon compensation for tourism in the tourism city agglomeration of the Yellow River Basin; give play to the leading role of provinces with high efficiency in tourism carbon emission reduction; realize the integration and interoperability of tourism carbon emission reduction technologies, concepts, and management in the Yellow River Basin; improve the efficiency of tourism carbon emission reduction in surrounding areas; and use carbon emission reduction as a link to realize a regional new governance model for coordinated economic development.

## Data Availability

The data that support the findings of this study are available in China National Basic Geographic Information Center at http://www.ngcc.cn. These data were derived from the following resources available in the public domain: Geospatial data cloud (https://www.gscloud.cn/); Data Center for Resources and Environmental Sciences, CAS (http://www.resdc.cn); China City Statistical Yearbook and Statistical communiques of National Economic and Social Development of provinces and cities.
